# Breastfeeding in Infancy in Relation to Subsequent Physical Size: A 20-year Follow-up of the Ibaraki Children’s Cohort Study (IBACHIL)

**DOI:** 10.2188/jea.JE20200562

**Published:** 2023-02-05

**Authors:** Mizuki Sata, Kazumasa Yamagishi, Toshimi Sairenchi, Fujiko Irie, Keiko Sunou, Hiroshi Watanabe, Hiroyasu Iso, Hitoshi Ota

**Affiliations:** 1 Department of Preventive Medicine and Public Health, Keio University School of Medicine, Tokyo, Japan; 2 Ibaraki Health Plaza, Ibaraki, Japan; 3 Department of Public Health Medicine, Faculty of Medicine, and Health Services Research and Development Center, University of Tsukuba, Ibaraki, Japan; 4 Ibaraki Health Service Association, Ibaraki, Japan; 5 Department of Public Health, Dokkyo Medical University School of Medicine, Tochigi, Japan; 6 Department of Health and Welfare, Ibaraki Prefectural Office, Ibaraki, Japan; 7 Faculty of Human Science, Tokiwa University, Ibaraki, Japan; 8 Public Health, Department of Social Medicine, Osaka University Graduate School of Medicine, Osaka, Japan

**Keywords:** children, body mass index, breast feeding, cohort study, epidemiology

## Abstract

**Background:**

Breastfeeding is said to prevent overweight and obesity in childhood but the evidence about its long-term impact on body size into adolescence and adulthood is scarce. We sought to examine the association between feeding types and subsequent physical size at the ages of 3, 6, 12, and 22 years.

**Methods:**

The Ibaraki Children’s Cohort (IBACHIL) Study, which began in 1992, involved a cohort of 4,592 Japanese children from 87 communities of a single prefecture whose parents answered health questionnaires about their child’s health and life habits at the age of 3 years. Follow-up questionnaires were distributed to the same cohort when they were 6, 12, and 22 years old. Self-reported height and weight, body mass index (BMI), and overweight status at ages of 3 (*n* = 4,290), 6 (*n* = 1,999; proportion of participants analyzed = 47%), 12 (*n* = 2,227; 52%), and 22 (*n* = 1,459; 34%) years were compared according to feeding type (breastfeeding, formula feeding, and mixed feeding) during infancy.

**Results:**

At the age of 3 years, multivariable adjusted-mean weight and prevalence of overweight were less for breastfed children than those formula-fed in both boys (weight: 14.6 kg vs 14.7 kg, *P* = 0.07, overweight: 6.3% vs 9.3%, *P* = 0.03) and in girls (14.0 kg vs 14.2 kg, *P* = 0.01 and 10.4% vs 13.6%, *P* = 0.06). However, there were no statistically significant differences in weight, BMI, and overweight at the ages of 6, 12, and 22 years according to feeding type.

**Conclusion:**

Breastfeeding may prevent overweight in childhood, but its impact is not significant in adolescence and adulthood.

## INTRODUCTION

The World Health Organization has recommended that babies be breastfed for the first 6 months of life.^1^ A systematic review (including cohort, case-control, and cross-sectional studies) reported that infants fed on breast milk had a reduced risk of childhood obesity (pooled odds ratio [OR] 0.74; 95% confidence interval [CI], 0.70–0.78).^2^ However, it has been less clear whether that effect would persist later in life; there have been only two cohort studies out of 12 published that reported a significant association between breastfeeding and subsequent overweight/obesity among ages 20 years or older.^2^ A quantitative review reported that whether feeding type-associated differences of mean body mass index (BMI; kg/m^2^) would remain long term was likely to be influenced by publication bias and confounding factors.^3^ Therefore, we sought to systematically investigate the long-term effects of feeding types during infancy on height, weight, BMI, and overweight by surveying a Japanese cohort at multiple time points (3, 6, 12, and 22 years old) and adjusting for environmental factors, such as parental occupation and parental physical size.

## METHODS

### Participants

The Ibaraki Children’s Cohort Study (IBACHIL) is a birth cohort study involving children born in 1989 from 87 communities across Ibaraki Prefecture, Japan. In 1992, a health questionnaire was distributed to parents of children who attended a community-based health check for 3-year-olds. Follow-up questionnaire surveys were carried out when the children were at the ages of 6 and 12 years and when they reached early adulthood at 22. The questionnaire surveys were completed by the parents of the children at ages 3, 6, and 12 years, but at age 22, the young adults themselves completed the survey.

The study protocol was approved by the Epidemiology Combination Ethic Review Committee of Ibaraki Prefecture.

### Baseline measurements

The parent-administrated questionnaire, distributed when the child was 3 years old, included several items related to lifestyle and physical factors, such as height, weight, eating-habits (including feeding in infancy), and living arrangement. The participants’ heights and weights were transcribed from medical records of their health checkup for 3-year-olds and recorded in their Maternal and Child Health Handbook (MCH Handbook) by qualified medical professionals in clinical settings.^4^ Types of feeding were classified as breastfed, formula-fed, or mixed fed.

Baseline BMI was calculated as weight (kg) divided by the square of the height in meters (m^2^). Overweight was defined as BMI ≥17.89 kg/m^2^ for boys and ≥17.56 kg/m^2^ for girls.^5^ “Waking up after 9 am” was defined as waking up late, “going to bed after 11 pm” as going to bed late, “outdoor play” as playing outside, and “father’s/mother’s full- or part-time job” as father’s/mother’s employment. Parental BMI was calculated as weight (kg) divided by the square of the height in meters (m^2^). Parental overweight status was defined as BMI ≥25.0 kg/m^2^.

### Follow-up measurements

The follow-up questionnaire surveys carried out at ages 6, 12, and 22 years asked the respondents their height (cm) and weight (kg). Overweight was defined as BMI ≥17.55 kg/m^2^ for boys and ≥17.34 kg/m^2^ for girls at the age of 6 years; ≥21.22 kg/m^2^ for boys and ≥21.68 kg/m^2^ for girls at the age of 12 years; and ≥25.0 kg/m^2^ at the age of 22 years.^5^

### Statistical analyses

Differences in mean values or frequencies of the baseline characteristics that were assumed to be potential confounding factors in the present study, according to the feeding type (breastfed, formula-fed, and mixed fed) were tested using the analysis of variance. Sex-specific mean values and proportions for each outcome according to the feeding types were examined using the analysis of covariance, adjusted for the participant’s baseline paternal and maternal height, weight, and occupation as is related to feeding type. To examine potential follow-up bias, baseline characteristics between those who dropped out and those who continued follow-up at ages of 6, 12, and 22 years (eTable 1, eTable 2, and eTable 3) were compared using the Student *t*-test or chi-square analysis. Baseline characteristics were also investigated using analysis of variance according to feeding type any by the participants’ age (6, 12, and 22 years old) (eTable 4, eTable 5, and eTable 6).

All statistical analyses were performed with SAS version 9.4 software (SAS Institute, Inc., Cary, NC, USA). All probability values for statistical tests were two-tailed, and *P* values of <0.05 were regarded as statistically significant.

## RESULTS

Among 29,375 children born in Ibaraki Prefecture in 1989,^6^ parents of 4,592 children returned completed questionnaires when they were 3 years old; 2,141 returned the follow-up questionnaire at age 6 (follow-up rate, 46.6%); 2,375 at age 12 (51.7%); and 1,559 participants returned the follow-up questionnaire at age 22 (34.0%). A total of 302 participants whose feeding information was incomplete at the baseline (3 years old) questionnaire, whose gender was undetermined, or who were outliers in terms of birthweight (low birth weight: <2,500 g or high birth weight: ≥5,000 g) were excluded from the study. The final analyses included 4,290 children (2,032 girls and 2,258 boys) at the age of 3 years; 1,999 children (938 girls and 1,061 boys) at the age of 6; 2,227 children (993 girls and 1,234 boys) at the age of 12; and 1,459 young adults (688 women and 771 men) at the age of 22.

The distribution of nutrient feeding frequency was: 30.7% (29.4% in boys and 32.2% in girls) for breastfed, 40.8% (41.9% in boys and 39.6% in girls) for formula-fed, and 28.5% (28.7% in boys and 28.3% in girls) for mixed fed. As shown in Table 1, breastfed children were more likely to have brothers or sisters. Fathers and mothers of breastfed boys had higher mean height, and their mothers had lower mean weight and BMI, and there was a lower proportion of overweight. Mixed-fed boys’ mothers had lower mean weight and BMI and there was a lower proportion of overweight. There was no association between feeding types and girls’ paternal physical size. The proportion of employed mothers was higher among formula-fed children than breastfed children. Other characteristics did not differ across the feeding types.

**Table 1.** Sex-specific mean values (standard deviations) and proportions of baseline characteristics at age of 3 years among 2,258 boys and 2,032 girls, IBACHIL

Types of feeding	Boys			Girls		
	
	Formula feeding	Mixed feeding	Breast feeding	Formula feeding	Mixed feeding	Breast feeding
	(*n* = 946)	(*n* = 648)	(*n* = 664)	(*n* = 804)	(*n* = 574)	(*n* = 654)
Birth height, cm	50.0 (1.9)	50.1 (1.8)	50.1 (1.7)	49.4 (1.8)	49.4 (1.8)	49.5 (1.9)
Birth weight, kg	3.2 (0.4)	3.3 (0.4)	3.3 (0.4)	3.1 (0.4)	3.2 (0.4)	3.2 (0.3)
Having brothers or sisters, %	74.9	78.9	82.7^***^	74.8	75.4	81.8^**^
Waking up late ≥9 am, %	4.8	3.4	4.1	7.1	5.4	4.7
Sleeping late ≥11 pm, %	5.7	4.9	5.3	5.5	7.3	4.9
Active body movements, %	90.4	92.7	92.8	89.8	90.7	88.5
Playing outside, %	94.9	94.6	95.3	93.9	95.1	95.0
Paternal height, cm	170.3 (5.6)	170.7 (5.6)	171.0 (5.5)^**^	170.1 (5.6)	170.3 (5.4)	170.5 (5.7)
Paternal weight, kg	66.8 (9.4)	67.7 (9.7)	67.5 (9.1)	66.9 (9.1)	67.1 (9.1)	67.4 (9.3)
Paternal body mass index, kg/m^2^	23.0 (2.9)	23.2 (2.9)	23.1 (2.8)	23.1 (2.7)	23.1 (2.8)	23.2 (2.8)
Paternal overweight, %	22.4	24.2	23.2	21.1	25.5	23.9
Father employed, %	81.4	83.3	83.6	81.7	81.0	82.6
Maternal height, cm	157.0 (5.2)	157.4 (5.0)	157.6 (4.9)^*^	157.0 (4.8)	157.3 (4.9)	157.4 (4.8)
Maternal weight, kg	53.2 (7.9)	52.3 (6.6)^*^	52.2 (6.1)^**^	52.4 (6.8)	52.3 (6.7)	52.1 (6.7)
Maternal body mass index, kg/m^2^	21.5 (2.9)	21.1 (2.4)^***^	21.0 (2.2)^***^	21.3 (2.6)	21.2 (2.6)	21.0 (2.5)
Maternal overweight, %	11.4	6.4^***^	5.7^***^	8.5	8.3	7.2
Mother employed, %	24.8	24.1	13.7^***^	25.2	26.3	13.8^***^

^*^P < 0.05, ^**^P < 0.01, ^***^P < 0.001, compared with formula feeding tested using the analysis of variance.

At the age of 3 years, multivariable-adjusted mean height and weight were lower among breastfed boys and girls than the formula-fed (Table 2). Mean BMI was less for breastfed girls than those who were formula-fed. The proportion of overweight was less for breastfed boys and girls than the formula-fed (boys: 6.3% vs 9.3%, *P* = 0.03 and girls: 10.4% vs 13.6%, *P* = 0.06). At the ages of 6, 12, and 22 years, no such differences were observed for both boys and girls.

**Table 2.** Multivariable-adjusted mean values (standard errors) and proportions of physical size according to the types of feeding at ages of 3, 6, 12, and 22 years

Types of feeding	Boys			Girls		
	
	Formula feeding	Mixed feeding	Breast feeding	Formula feeding	Mixed feeding	Breast feeding
Age of 3 years						
*n*	946	648	664	804	574	654
Height, cm	95.0 (0.11)	95.0 (0.13)	94.6 (0.13)^**^	93.7 (0.11)	93.8 (0.13)	93.3 (0.12)^*^
Weight, kg	14.7 (0.05)	14.8 (0.06)	14.6 (0.06)^†^	14.2 (0.06)	14.2 (0.07)	14.0 (0.06)^*^
Body mass index, kg/m^2^	16.3 (0.04)	16.3 (0.05)	16.3 (0.05)	16.2 (0.04)	16.1 (0.05)	16.0 (0.05)^†^
Overweight, %	9.3	8.7	6.3^*^	13.6	10.9	10.4^†^

Age of 6 years						
*n*	443	287	331	367	267	304
Height, cm	114.0 (0.24)	113.7 (0.28)	114.0 (0.27)	112.5 (0.27)	112.9 (0.32)	113.2 (0.30)^†^
Weight, kg	20.8 (0.18)	20.7 (0.21)	21.1 (0.20)	20.2 (0.18)	20.4 (0.21)	20.4 (0.20)
Body mass index, kg/m^2^	15.9 (0.10)	16.0 (0.12)	16.2 (0.11)	15.9 (0.10)	16.0 (0.12)	15.9 (0.11)
Overweight, %	12.7	10.7	17.1	15.9	16.2	16.0

Age of 12 years						
*n*	520	337	377	407	269	317
Height, cm	155.2 (0.34)	155.7 (0.42)	155.1 (0.40)	153.0 (0.27)	153.5 (0.34)	153.4 (0.31)
Weight, kg	46.2 (0.41)	46.1 (0.51)	46.0 (0.48)	44.3 (0.38)	45.2 (0.47)	45.0 (0.44)
Body mass index, kg/m^2^	19.1 (0.13)	18.9 (0.16)	19.0 (0.15)	18.9 (0.13)	19.1 (0.17)	19.0 (0.15)
Overweight, %	19.9	15.1^†^	18.4	14.5	14.6	14.6

Age of 22 years						
*n*	306	222	243	283	191	214
Height, cm	171.9 (0.30)	171.8 (0.35)	172.5 (0.34)	158.6 (0.27)	158.1 (0.33)	158.6 (0.31)
Weight, kg	64.4 (0.56)	63.5 (0.65)	65.4 (0.63)	51.9 (0.44)	51.8 (0.54)	51.4 (0.51)
Body mass index, kg/m^2^	21.8 (0.18)	21.5 (0.21)	21.9 (0.20)	20.6 (0.16)	20.7 (0.20)	20.4 (0.19)
Overweight, %	13.4	10.4	13.9	7.6	6.8	4.9

^†^*P* < 0.1, ^*^P < 0.05, ^**^P < 0.01, compared with formula feeding tested using the analysis of covariance.

Adjusted for paternal and maternal height, weight, and occupation.

We compared the baseline characteristics, including the distribution of feeding types, between those who dropped out and those who continued follow-up at ages of 6, 12, and 22 years. There were no material differences regarding children’s height, weight, BMI, and overweight status at age of 3 years between participants and non-participants (eTable 1, eTable 2, and eTable 3). As shown in eTable 4, eTable 5, and eTable 6, we observed similar baseline characteristics according to the feeding types among participants at ages of 6, 12, and 22 years.

## DISCUSSION

We found that, at age of 3 years, boys and girls who were breastfed in infancy were smaller in stature and lighter in body weight than those who were formula-fed. The proportion of overweight children was lower for breastfed girls and boys than those formula-fed; however, no such associations were found at ages of 6, 12, and 22 years.

The association of feeding types with subsequent body size and obesity has been reported from many studies of infants,^2^^,^^7^^–^^9^ several studies^10^^,^^11^ of school children, and to our best knowledge, only two studies^12^^,^^13^ of adults. In a birth cohort of 1,265 children born in Christchurch, New Zealand in 1977, a significant association was found between longer duration of breast feeding and lower adult BMI at ages 30 and 35.^12^ Another cohort study of 9,377 children born in England, Scotland, and Wales in 1958 reported that breastfeeding for more than 1 month was associated with lower risk of obesity (BMI ≥30 kg/m^2^) at ages of 44 to 45 years, compared with formula feeding (multivariable relative risk 0.85; 95% CI, 0.75–0.97).^13^ On the other hand, other cohort studies found no association between breastfeeding and risk of overweight/obesity in adulthood.^14^^,^^15^ In the present study, we found that the proportion of those overweight among 3-year-olds was lower for breastfed than formula-fed children, but these differences were not observed at ages 6, 12, and 22, suggesting that the potential benefit of breastfeeding is unlikely lasts for many years.

Though the mechanisms linking breastfeeding to childhood physical size are not clear, bioactive and behavioral factors are likely to play a role. Breastfeeding decreases appetite-associated peptides and secretion of ghrelin, which may reduce infant appetite, leading to reduced risk of obesity.^16^ Leptin, a hormone that regulates food intake and energy metabolism, is present in breast milk and serum leptin values have been reported to be higher in breastfed than in formula-fed infants.^17^ Formula-fed children have been reported to have a higher protein intake than breastfed children.^18^ Higher protein intake in early childhood is thought to upregulate secretion of insulin and insulin-like growth factor-1 (IGF-1), which are associated with an early adiposity rebound.^19^ Additionally, the capacity for self-regulation of milk intake may affect the infant’s body size. In a previous study, formula-fed infants in early infancy were more likely to empty the bottle in late infancy than were those who were fed directly at the breast, regardless of breast or formula milk.^20^

Our finding of no long-term effect of breastfeeding on adolescence or adulthood BMI was consistent with the results of previous studies.^3^ The long-term importance of breastfeeding on BMI may be limited because lifestyles at adolescence and adulthood may have a greater impact on BMI.

The strength of this study is its long-term prospective design. As far as we know, there have been no reports from Asia on the long-term effects of breastfeeding on children’s height, weight, and BMI from infancy into early adulthood.

On the other hand, the present study has several limitations. First, while the heights and weights of the 3-year-olds were accurately transcribed from medical records recorded in the participants’ MCH Handbooks, those of the follow-up surveys were derived from responses to self-administered questionnaires. A previous study of 490 girls and 385 boys aged 8–12 years showed that self-reported weight was understated by 1.255 kg for girls and 0.572 kg for boys, and height was also understated by 0.951 cm for girls and 0.655 cm for boys.^21^ In another study, involving 5,870 women and 2,200 men and aged 20–44 years, weight was understated by 0.8 kg for women and 0.3 kg for men, whereas height was overstated by 0.7 cm for women and 0.6 cm for men.^22^ However, any possible misclassification resulting from self-reporting should be non-differential according to feeding types; thus, the observed association in our study may be somewhat underestimated and the real associations could be stronger. Second, the follow-up rate was not very high (47% at age of 6 years, 52% at age of 12 years, and 34% at age of 22 years). The follow-up rate from baseline (birth to infancy) to adulthood were higher in England,^13^ New Zealand,^12^ Brazil,^23^ and the Philippines^24^ (78% at 44–45 years, 76% at 35 years, 73% at 23 years, and 62% at 21 years) but lower in India^25^ (19% at 32 years). However, we found that the baseline characteristics, including the distribution of feeding types, did not differ materially between participants and non-participants at ages of 6, 12, and 22 years. We also observed similar baseline characteristics according to feeding type among participants at ages of 6, 12, and 22 years. Therefore, the possibility of follow-up bias would be minimal. Third, we did not collect information on duration of breastfeeding and timing of solid foods introduction. Previous studies have reported that a longer duration of breastfeeding^8^ or delayed introduction of solids^26^ was associated with a decreased risk of being overweight. Fourth, we could not adjust for unmeasured confounders, such as socioeconomic status^27^ and maternal smoking status.^28^ However, the association between breastfeeding and a child’s subsequent body size did not change after adjustment for parental occupation, which is one of the major socioeconomic status variables. We did not collect data on maternal smoking status, but the prevalence of maternal smoking was reported low (5%) in Japan.^29^

### Conclusion

Breastfeeding may contribute to appropriate body size for girls and boys in early childhood, but its impact is diminished long-term in adolescence or adulthood; therefore, the beneficial effect of feeding types on subsequent children’s physical size may be limited to the first few years of life.

## SUPPLEMENTARY MATERIAL

eTable 1. Sex-specific mean values (standard deviations) and proportions of baseline characteristics at age of 6 years between participants and non-participants

eTable 2. Sex-specific mean values (standard deviations) and proportions of baseline characteristics at age of 12 years between participants and non-participants

eTable 3. Sex-specific mean values (standard deviations) and proportions of baseline characteristics at age of 22 years between participants and non-participants

eTable 4. Sex-specific mean values (standard deviations) and proportions of baseline characteristics among participants at age of 6 years

eTable 5. Sex-specific mean values (standard deviations) and proportions of baseline characteristics among participants at age of 12 years

eTable 6. Sex-specific mean values (standard deviations) and proportions of baseline characteristics among participants at age of 22 years
