# Supplementary material for: Breastfeeding in Infancy in Relation to Subsequent Physical Size: A 20-year Follow-up of the Ibaraki Children’s Cohort Study (IBACHIL)
Source: J Epidemiol. 2023 Feb 5;33(2):63–7. doi: 10.2188/jea.JE20200562 (PMC9794444; doi:10.2188/jea.JE20200562)
Supplement: Supplementary file 1 [file je-33-063-s001.pdf]

eTable 1. Sex-specific mean values (standard deviations) and proportions of baseline characteristics at age of 6 years between participants and non-participants

Types of feeding	Boys		Girls	
	Participants (n=1,061)	Non-Participants (n=1,197)	Participants (n=938)	Non-Participants (n=1,094)
Formula feeding, %	41.8	42.0	39.1	39.9
Mixed feeding, %	27.0	30.2	28.5	28.1
Breast feeding, %	31.2	27.8	32.4	32.0
Birth height, cm	50.0 (1.8)	50.1 (1.9)	49.4 (1.8)	49.5 (1.8)
Birth weight, kg	3.3 (0.4)	3.2 (0.4)	3.1 (0.3)	3.2 (0.3)
Height at age of 3 years	94.9 (3.5)	94.9 (3.6)	93.5 (3.4)	93.7 (3.5)
Weight at age of 3 years	14.7 (1.6)	14.7 (1.7)	14.1 (1.6)	14.2 (1.7)
Body mass index at age of 3 years	16.3 (1.2)	16.3 (1.3)	16.1 (1.2)	16.1 (1.3)
Overweight at age of 3 years	8.1	8.4	11.6	12.0
Having brothers or sisters, %	78.0	78.7	77.7	76.8
Waking up late ≥9 am, %	4.2	4.1	6.5	5.3
Sleeping late ≥11 pm, %	5.0	5.7	6.3	5.4
Active body movements, %	90.7	92.7	88.9	89.8
Playing outside, %	94.9	95.0	94.5	94.7
Paternal height, cm	170.5 (5.6)	170.7 (5.5)	170.1 (5.5)	170.5 (5.6)
Paternal weight, kg	67.1 (9.4)	67.4 (9.4)	67.3 (9.2)	66.9 (9.2)
Paternal body mass Index, kg/m^2	23.1 (2.8)	23.1 (2.9)	23.3 (2.8)	23.0 (2.7)
Paternal overweight, %	22.7	23.5	25.1	21.6
Father employed, %	84.1	81.3	81.0	82.4
Maternal height, cm	157.1 (5.0)	157.5 (5.1)	157.3 (5.0)	157.1 (4.8)
Maternal weight, kg	52.6 (6.8)	52.6 (7.2)	52.3 (6.6)	52.3 (6.9)
Maternal body mass Index, kg/m^2	21.3 (2.6)	21.2 (2.6)	21.1 (2.5)	21.2 (2.6)
Maternal overweight, %	8.3	8.2	7.3	8.6
Mother employed, %	21.1	21.6	22.3	21.5

**P*<0.05, ***P*<0.01, ****P*<0.001, compared with participants, tested using the Student t-test or chi-square analysis

"Non-participants" refers to those who dropped out of the study at the age of 6 years.

eTable 2. Sex-specific mean values (standard deviations) and proportions of baseline characteristics at age of 12 years between participants and non-participants

	Boys		Girls	
Types of feeding	Participants (n=1,234)	Non-Participants (n=1,024)	Participants (n=993)	Non-Participants (n=1,039)
Formula feeding, %	42.1	41.6	41.0	38.2
Mixed feeding, %	27.3	30.4	27.1	29.4
Breast feeding, %	30.6	28.0	31.9	32.4
Birth height, cm	50.1 (1.8)	50.1 (1.9)	49.4 (1.9)	49.4 (1.8)
Birth weight, kg	3.3 (0.4)	3.2 (0.4)	3.2 (0.4)	3.1 (0.3)
Height at age of 3 years	94.9 (3.5)	94.9 (3.6)	93.6 (3.3)	93.5 (3.5)
Weight at age of 3 years	14.7 (1.6)	14.7 (1.8)	14.1 (1.6)	14.1 (1.7)
Body mass index at age of 3 years	16.3 (1.2)	16.3 (1.3)	16.1 (1.2)	16.1 (1.3)
Overweight at age of 3 years	7.9	8.7	12.2	11.5
Having brothers or sisters, %	80.6	75.7**	81.6	73.1***
Waking up late ≥9 am, %	3.6	4.9	5.2	6.4
Sleeping late ≥11 pm, %	4.4	6.5*	4.9	6.6
Active body movements, %	91.8	91.7	90.1	88.6
Playing outside, %	94.4	95.6	96.1	93.2*
Paternal height, cm	170.5 (5.6)	170.8 (5.6)	170.1 (5.5)	170.5 (5.6)
Paternal weight, kg	67.1 (9.3)	67.4 (9.5)	67.1 (8.8)	67.1 (9.5)
Paternal body mass Index, kg/m^2	23.1 (2.8)	23.1 (3.0)	23.2 (2.7)	23.1 (2.8)
Paternal overweight, %	23.0	23.3	22.9	23.6
Father employed, %	82.7	82.4	80.2	83.3
Maternal height, cm	157.0 (5.0)	157.6 (5.1)**	157.2 (4.9)	157.2 (4.9)
Maternal weight, kg	52.5 (6.9)	52.8 (7.2)	52.2 (6.5)	52.4 (7.0)
Maternal body mass Index, kg/m^2	21.3 (2.5)	21.2 (2.7)	21.1 (2.5)	21.2 (2.7)
Maternal overweight, %	7.8	8.8	7.7	8.3
Mother employed, %	24.6	17.5***	24.4	19.4**

*P<0.05, **P<0.01, ***P<0.001, compared with participants, tested using the Student t-test or chi-square analysis

"Non-participants" refers to those who dropped out of the study at the age of 12 years.

eTable 3. Sex-specific mean values (standard deviations) and proportions of baseline characteristics at age of 22 years between participants and non-participants

Types of feeding	Boys		Girls	
	Participants (n=771)	Non-Participants (n=1,487)	Participants (n=688)	Non-Participants (n=1,344)
Formula feeding, %	39.7	43.0	41.1	38.8
Mixed feeding, %	28.8	28.6	27.8	28.5
Breast feeding, %	31.5	28.3	31.1	32.7
Birth height, cm	50.1 (1.9)	50.1 (1.8)	49.4 (2.0)	49.4 (1.8)
Birth weight, kg	3.3 (0.4)	3.3 (0.4)	3.2 (0.3)	3.2 (0.4)
Height at age of 3 years	95.0 (3.4)	94.9 (3.6)	93.7 (3.4)	93.5 (3.5)
Weight at age of 3 years	14.7 (1.5)	14.7 (1.8)	14.2 (1.6)	14.1 (1.7)
Body mass index at age of 3 years	16.3 (1.2)	16.3 (1.3)	16.2 (1.2)	16.1 (1.3)
Overweight at age of 3 years	7.7	8.6	12.2	11.6
Having brothers or sisters, %	81.6	76.7**	80.4	75.6*
Waking up late ≥9 am, %	3.2	4.6	5.8	5.9
Sleeping late ≥11 pm, %	3.9	6.1*	4.5	6.5
Active body movements, %	92.3	91.5	87.5	90.3
Playing outside, %	94.9	95.0	95.2	94.3
Paternal height, cm	170.4 (5.7)	170.7 (5.5)	170.2 (5.4)	170.4 (5.6)
Paternal weight, kg	66.8 (9.4)	67.5 (9.4)	67.1 (9.0)	67.1 (9.3)
Paternal body mass Index, kg/m^2	23.0 (2.8)	23.2 (3.0)	23.2 (2.7)	23.1 (2.8)
Paternal overweight, %	21.1	24.2	23.1	23.3
Father employed, %	83.3	82.2	81.1	82.1
Maternal height, cm	157.1 (5.1)	157.4 (5.1)	157.2 (4.9)	157.2 (4.9)
Maternal weight, kg	52.4 (6.7)	52.7 (7.2)	52.4 (6.7)	52.2 (6.8)
Maternal body mass Index, kg/m^2	21.2 (2.4)	21.3 (2.7)	21.2 (2.5)	21.1 (2.6)
Maternal overweight, %	7.6	8.6	8.2	7.9
Mother employed, %	24.8	19.6**	24.1	20.7

*P<0.05, **P<0.01, ***P<0.001, compared with participants, tested using the Student t-test or chi-square analysis

"Non-participants" refers to those who dropped out of the study at the age of 22 years.

eTable 4. Sex-specific mean values (standard deviations) and proportions of baseline characteristics among participants at age of 6 years

	Boys			Girls		
Types of feeding	Formula feeding (n=443)	Mixed feeding (n=287)	Breast feeding (n=331)	Formula feeding (n=367)	Mixed feeding (n=267)	Breast feeding (n=304)
Birth height, cm	50.0 (1.9)	50.2 (1.8)	50.0 (1.7)	49.2 (1.7)	49.3 (1.9)	49.6 (1.9)**
Birth weight, kg	3.2 (0.4)	3.3 (0.4)	3.3 (0.4)	3.1 (0.3)	3.2 (0.4)*	3.2 (0.3)*
Height at age of 3 years, cm	94.9 (3.5)	95.1 (3.3)	94.7 (3.5)	93.4 (3.3)	93.7 (3.5)	93.4 (3.3)
Weight at age of 3 years, kg	14.7 (1.6)	14.8 (1.6)	14.6 (1.6)	14.1 (1.6)	14.2 (1.6)	14.0 (1.6)
Body mass index at age of 3 years, kg/m^2	16.3 (1.2)	16.3 (1.2)	16.2 (1.1)	16.2 (1.2)	16.1 (1.2)	16.0 (1.2)
Overweight at age of 3 years, %	9.3	8.9	5.8	13.3	9.6	11.5
Having brothers or sisters, %	73.1	79.1	83.4***	77.7	73.4	81.6
Waking up late ≥9 am, %	5.2	3.5	3.6	6.8	6.4	6.3
Sleeping late ≥11 pm, %	5.2	4.2	5.4	5.7	7.9	5.6
Active body movements, %	89.4	92.7	90.6	89.1	90.6	87.2
Playing outside, %	92.6	96.2*	97.0**	94.0	94.0	95.4
Paternal height, cm	170.2 (5.5)	170.4 (6.0)	171.1 (5.4)*	170.1 (5.4)	169.8 (5.6)	170.4 (5.6)
Paternal weight, kg	66.3 (9.4)	68.2 (10.1)**	67.4 (8.7)	66.9 (8.8)	67.3 (9.5)	67.8 (9.3)
Paternal body mass Index, kg/m^2	22.8 (2.9)	23.5 (2.9)**	23.0 (2.7)	23.1 (2.7)	23.3 (2.9)	23.3 (2.8)
Paternal overweight, %	20.3	27.2*	22.0	21.1	28.3*	27.2
Father employed, %	83.5	87.1	82.2	82.3	80.5	79.9
Maternal height, cm	156.9 (5.2)	157.1 (4.8)	157.4 (4.9)	157.1 (4.9)	157.5 (5.1)	157.2 (4.9)
Maternal weight, kg	52.8 (7.6)	52.8 (6.7)	52.2 (5.8)	52.3 (6.7)	52.4 (6.5)	52.2 (6.5)
Maternal body mass Index, kg/m^2	21.4 (2.8)	21.4 (2.5)	21.1 (2.1)*	21.2 (2.5)	21.1 (2.4)	21.1 (2.5)
Maternal overweight, %	11.0	8.1	4.9**	8.0	6.8	7.0
Mother employed, %	27.1	22.3	12.1***	25.3	27.3	14.1***

**P*<0.05, ***P*<0.01, ****P*<0.001, compared with formula feeding, tested using the analysis of variance

eTable 5. Sex-specific mean values (standard deviations) and proportions of baseline characteristics among participants at age of 12 years

Types of feeding	Boys			Girls		
	Formula feeding (n=520)	Mixed feeding (n=337)	Breast feeding (n=377)	Formula feeding (n=407)	Mixed feeding (n=269)	Breast feeding (n=317)
Birth height, cm	50.0 (1.8)	50.1 (1.8)	50.1 (1.7)	49.4 (1.8)	49.4 (1.9)	49.6 (2.1)
Birth weight, kg	3.2 (0.4)	3.3 (0.4)	3.3 (0.4)	3.1 (0.4)	3.2 (0.4)	3.2 (0.3)
Height at age of 3 years, cm	94.9 (3.5)	94.9 (3.5)	94.8 (3.4)	93.6 (3.4)	93.9 (3.4)	93.4 (3.2)
Weight at age of 3 years, kg	14.7 (1.7)	14.8 (1.6)	14.6 (1.5)	14.2 (1.6)	14.2 (1.6)	14.0 (1.5)
Body mass index at age of 3 years, kg/m^2	16.3 (1.3)	16.4 (1.2)	16.2 (1.1)	16.2 (1.3)	16.1 (1.3)	16.0 (1.2)*
Overweight at age of 3 years, %	9.6	8.4	5.1*	14.4	11.5	10.0
Having brothers or sisters, %	76.9	81.6	84.6**	80.8	79.6	84.2
Waking up late ≥9 am, %	4.4	3.0	2.9	5.4	3.7	6.3
Sleeping late ≥11 pm, %	4.2	3.6	5.3	4.2	5.9	5.0
Active body movements, %	90.4	93.5	92.3	91.4	90.3	88.3
Playing outside, %	93.8	94.7	95.0	95.3	95.9	97.2
Paternal height, cm	170.2 (5.6)	170.4 (5.5)	171.0 (5.6)*	170.0 (5.6)	170.0 (5.2)	170.4 (5.7)
Paternal weight, kg	66.5 (9.3)	67.7 (9.7)	67.4 (9.0)	66.5 (8.9)	67.3 (8.6)	67.7 (9.0)
Paternal body mass Index, kg/m^2	22.9 (2.8)	23.3 (2.9)	23.1 (2.8)	23.0 (2.7)	23.3 (2.7)	23.3 (2.8)
Paternal overweight, %	21.9	25.2	22.5	19.8	26.7*	23.7
Father employed, %	83.3	83.4	81.4	82.3	80.7	77.0
Maternal height, cm	156.7 (5.2)	157.2 (4.8)	157.3 (5.0)*	157.0 (5.0)	157.4 (4.9)	157.2 (4.6)
Maternal weight, kg	52.6 (7.6)	52.5 (6.6)	52.3 (6.1)	52.4 (6.6)	52.5 (6.4)	51.8 (6.4)
Maternal body mass Index, kg/m^2	21.4 (2.8)	21.3 (2.4)	21.1 (2.2)	21.2 (2.5)	21.2 (2.4)	20.9 (2.4)
Maternal overweight, %	10.2	6.3*	6.0*	8.2	8.0	6.7
Mother employed, %	29.0	27.6	15.6***	27.8	30.9	14.5***

*P<0.05, **P<0.01, ***P<0.001, compared with formula feeding, tested using the analysis of variance

eTable 6. Sex-specific mean values (standard deviations) and proportions of baseline characteristics among participants at age of 22 years

Types of feeding	Boys			Girls		
	Formula feeding (n=306)	Mixed feeding (n=222)	Breast feeding (n=243)	Formula feeding (n=283)	Mixed feeding (n=191)	Breast feeding (n=214)
Birth height, cm	50.0 (1.9)	50.1 (1.9)	50.2 (1.7)	49.3 (1.9)	49.5 (1.8)	49.4 (2.2)
Birth weight, kg	3.2 (0.4)	3.3 (0.4)	3.3 (0.4)*	3.1 (0.3)	3.2 (0.3)	3.2 (0.3)
Height at age of 3 years, cm	95.0 (3.5)	94.9 (3.1)	94.9 (3.5)	93.8 (3.4)	93.9 (3.3)	93.5 (3.3)
Weight at age of 3 years, kg	14.7 (1.5)	14.8 (1.5)	14.6 (1.5)	14.2 (1.6)	14.3 (1.5)	14.1 (1.5)
Body mass index at age of 3 years, kg/m^2	16.2 (1.2)	16.4 (1.1)*	16.2 (1.2)	16.2 (1.3)	16.2 (1.1)	16.1 (1.2)
Overweight at age of 3 years, %	7.9	9.6	5.5	14.1	10.2	11.5
Having brothers or sisters, %	77.8	81.5	86.4**	75.6	82.7	84.6*
Waking up late ≥9 am, %	3.9	3.2	2.5	6.0	5.2	6.1
Sleeping late ≥11 pm, %	3.6	3.6	4.5	5.3	5.8	2.3
Active body movements, %	92.5	93.7	90.9	88.3	88.0	86.0
Playing outside, %	94.8	93.7	96.3	94.3	95.3	96.3
Paternal height, cm	170.3 (5.7)	170.2 (5.8)	170.7 (5.8)	170.1 (5.3)	170.1 (5.1)	170.5 (6.0)
Paternal weight, kg	66.6 (9.7)	67.1 (9.9)	66.7 (8.5)	66.5 (8.6)	67.2 (8.7)	67.9 (9.8)
Paternal body mass Index, kg/m^2	22.9 (2.9)	23.2 (2.8)	22.9 (2.6)	23.0 (2.7)	23.2 (2.7)	23.3 (2.8)
Paternal overweight, %	20.1	23.0	20.7	18.9	27.8*	24.6
Father employed, %	83.3	86.5	80.2	83.7	80.6	78.0
Maternal height, cm	156.7 (5.2)	157.1 (4.8)	157.5 (5.2)	157.2 (4.9)	157.4 (5.2)	157.0 (4.4)
Maternal weight, kg	52.2 (7.3)	52.6 (6.4)	52.4 (6.1)	52.1 (6.8)	52.7 (6.1)	52.6 (7.0)
Maternal body mass Index, kg/m^2	21.2 (2.7)	21.3 (2.3)	21.1 (2.2)	21.1 (2.6)	21.3 (2.3)	21.3 (2.6)
Maternal overweight, %	10.2	6.0	5.9	7.5	8.0	9.5
Mother employed, %	29.7	28.8	14.8***	25.8	30.9	15.9*

**P*<0.05, ***P*<0.01, ****P*<0.001, compared with formula feeding, tested using the analysis of variance
